# Low-Cost Approach to an Instream Water Depth Sensor Construction Using Differential Pressure Sensors and Arduino Microcontrollers

**DOI:** 10.3390/s24082488

**Published:** 2024-04-12

**Authors:** Reagan H. Pearce, Michael A. Chadwick, Bruce Main, Kris Chan, Carl D. Sayer, Ian R. Patmore

**Affiliations:** 1 Department of Geography, Faculty of Social and Historical Sciences, University College London, Gower Street, London WC1E 6BT, UK; c.sayer@ucl.ac.uk (C.D.S.); i.patmore@ucl.ac.uk (I.R.P.); 2 Department of Geography, Faculty of Social Science and Public Policy, King’s College London, Strand, London WC2B 4BG, UK; michael.chadwick@kcl.ac.uk (M.A.C.); kristofer.chan@kcl.ac.uk (K.C.); 3 Lincoln University, 85084 Ellesmere Junction Road, Lincoln 7647, New Zealand; bruce.main@lincoln.ac.nz

**Keywords:** Arduino, low cost, monitoring, environmental sensors, hydrology, water depth

## Abstract

Accurate hydrological data with high spatial resolution is important for flood risk and water resource management, particularly under the context of climate change. The cost of monitoring networks, as well as the characteristics of the hydrological environment itself, can be a barrier to meeting these data requirements, however. This study covers the design and testing of a low-cost, “build-it-yourself”, instream water depth sensor providing an assessment of its potential in future hydrological monitoring projects. The low-cost sensor was built using an Arduino microcontroller, a differential pressure sensor and a thermistor, a real-time clock, and an SD card module. The low-cost logger was deployed in tandem with a factory-calibrated Solinst^®^LevelLogger^®^ 5 Junior for 6 months in the River Wissey, UK. We found the mean absolute error of the Arduino-based logger relative to the commercial setup to be ±0.69 cm for water depth and ±0.415 °C for water temperature. Economically, the Arduino-based logger offers an advantage, costing a total of £133.35 (USD 168.26 at time of publication) comparative to the industrial comparison’s cost of £408 (USD 514.83 at time of publication). This study concludes that the low cost of the Arduino-based logger gives a strong advantage to its incorporation in hydrological data collection, if the trade-offs (i.e., time investment and accuracy) are considered acceptable and appropriate for a project.

## 1. Introduction

Accurate, high-resolution hydrological data are the key to policy making across multiple disciplines, including flood risk analysis, water resource management, and climate change, where increased winter rainfall in some areas of the UK increases potential future flood risk [1]. In the UK, hydrological data, including water discharge, velocity, and depth, are collected in river channels [2]. The principal data portal is the National River Flow Archive (NFRA), comprised of approximately 1550 gauging stations (some records extending to the 1960s), which combined with extensive research studies, has consolidated the river monitoring record in the UK [3]. The gauging stations, however, have varying record lengths and uneven spatial distributions, resulting in some spatial and temporal gaps. In addition, these stations do not collect water temperature data, which is key to accurately constraining evapotranspiration causes of meteorological droughts and broader biochemical water conditions. Further, the UK monitoring network is slowly deteriorating, with a slow reduction in gauging stations (1559 peak in 2012, 1553 at present day) and a reduction in effective operation of existing stations. Ultimately, as data collection continues to decrease, local communities, conservation agencies and government are becoming increasingly dependent on the scientific community or private companies to conduct research and monitoring. Given the expense of equipment and installation structures, projects aimed at monitoring hydrology for flood risk evaluation, landscape-scale conservation planning, or long-term monitoring of climate change impacts need sufficient financial backing to achieve project objectives. Hence, while there is clearly a requirement for the increased collection of fine-scale hydrological data, this needs to occur at a low financial and resource cost. 

Low-cost, “build-it-yourself” sensors are increasingly popular due to the growing accessibility of open-source hardware, such as microcontrollers and sensors, and easy-to-code software that allows an individual to design, construct, and code their own environmental monitoring sensor [4,5,6,7]. The Maker movement [8] and the Internet of Things (IoT) [9] encourage non-experts to design and innovate with more accessible routes to technological tinkering by providing open-source software and hardware, troubleshooting through internet forums that fosters a sense of community, and resources such as instructions and video tutorials [10]. An increased accessibility of knowledge, as well as the low costs associated with training, equipment, and parts has resulted in an explosion of “build-it-yourself” engineering with a wide variety of applications. In the environmental sector, tinkerers have used open-source software and hardware to create sediment traps, water quality sensors, weather stations [6], spectrometers [11], and air quality sensors [7] at a fraction of the cost of commercial equivalents. Emerging research has shown, however, that there can be unexpected costs and pitfalls to applying open-source technology and a “build-it-yourself” approach to environmental monitoring [6]. The trial-and-error approach can increase labour hours and associated equipment costs. Moreover, the calibration of low-cost sensors is often less robust and results in less accurate data collection [6]. Where appropriate, however, low-cost, open-source sensors can provide an opportunity to gather high-resolution data both temporally and spatially at a reduced cost compared to equivalent factory-made equipment.

Key hydrological variables in river monitoring are discharge or flow, velocity, and water depth or stage. In combination with rainfall, topography, geology, and groundwater data, these variables are important to flood risk assessment and water resource management [12,13]. Hydrological variables are also key to understanding riverine ecology, such as fish habitat use, which is dependent on velocity and water depth [14,15,16,17]. Developments of space-borne, low-cost monitoring data has been essential in addressing multiple riverine monitoring issues, particularly with developing flood models [18], and now, low-cost sensor monitoring of hydrological variables can improve the spatial and temporal resolution of data collection.

Here, the design, construction, calibration and results from field testing comparisons against a factory-calibrated Solinst^®^ water level sensor are presented for a low-cost, instream water depth sensor deployed for six months in the River Wissey, eastern England. 

## 2. Materials and Methods

### 2.1. Water Depth Logger Design

**Figure 1:**
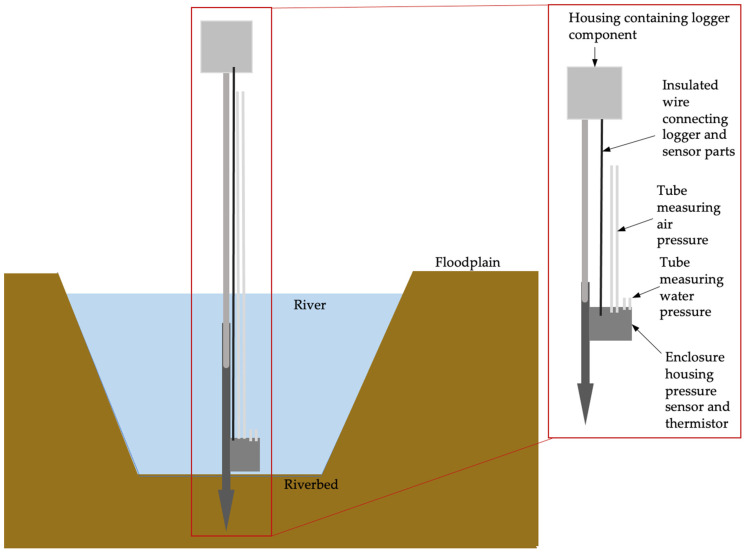
Concept for deployable water depth sensor using Arduino microcontroller and differential pressure sensor.

The sensor was designed to be as simple as possible in terms of construction and deployment. To produce a truly ‘low-cost’ sensor, parts were chosen based on their quality as well as their price (Table 1). More detailed instructions for construction are available at https://github.com/rhpearce/waterdepthsensor (accessed on 9 April 2024). Due to the challenges of waterproofing DIY sensors, the sensor design is orchestrated to have a submersible sensor part and a non-submersible housing part (Figure 1). The non-submersible part was elevated to beyond the known atypical extreme water level. In this deployment, the submersible sensor was held 2.5 m from the riverbed bottom, which was determined from winter aerial imagery and LiDAR data to identify the active floodplain as historical water level data were not available for the research area. To prevent water intrusion into the non-submersible housing, an enclosure with a high IP rating was chosen (RS PRO ABS General Purpose Enclosure, IP65, Light Grey; RS PRO, RS Components Ltd., Birchington Road, Corby, Northamptonshire NN17 9RS, UK, Manufactured in China). Here, the design was targeted for a wadeable, soft sediment riverbed, but could be adapted for other conditions depending on need. For example, for deeper rivers, the post could be attached to a concrete block to weigh it down at the bottom of the riverbed. For this deployment, however, the main logger housing was attached to a bracket (Angle aluminium, metals4U Ltd., Armitage Works, Sandbeck Way, Wetherby LS22 7DN, UK) which was screwed to a threaded bar (M10 steel threaded bar; Easyfix UK, Galway, Ireland), which was then attached to a sharpened angle iron base. Each angle iron base (metals4U Ltd, Armitage Works, Sandbeck Way, Wetherby LS22 7DN, UK) was cut based on the water depth measured in summer 2021, and an additional 50 cm was added to achieve anchorage in the sediment, which was mainly silt–sand and created its own stability due to the cohesive and adhesive forces between the water and sediment (see Section 2.5), comparative to a gravel or boulder bed stream. A parts list for deployment structure is listed in Table 2.

### 2.2. Key Components

#### 2.2.1. Arduino Microcontroller

As listed in Table 1, the Arduino Pro Mini 3.3 V (Kunkune Ltd., 100A High Street, Thame, Oxfordshire OX93EH, UK) was chosen as the microprocessor due to its low power consumption, compact design, and compatibility with the accessible Arduino IDE v2.2.1 (https://www.arduino.cc/en/software, accessed on 9 April 2024) platform. There is a vast array of literature on low-cost environmental sensors that use solely, or incorporate Arduino hardware, that support its choice as a microcontroller in this design [6]. 

#### 2.2.2. Differential Pressure Sensors

A differential pressure sensor was chosen to streamline the data collection process in which water pressure needs to be compensated by local air pressure. By using a differential pressure sensor, an immediate and locally accurate reading of water depth can be attained in the field without post-deployment processing applying proximate pressure readings. The 50 kPa differential sensor (MPX5050DP; NXP, High Tech Campus 60, 5656 AG, Eindhoven, Netherlands) is appropriate for use in small streams, with a maximum reading at 5098.72 mm H_2_O (~5.09 m), after which it does not give a measurement. The maximum pressure it can withstand is 200 kPa. Other capacity sensors are available, e.g., sensors up to 2000 kPa, or temperature compensated sensors, e.g., MPX2010DP. Adapting the circuitry to more powerful sensors will increase voltage requirements and power consumption, meaning a higher power microcontroller, such as the Arduino UNO (https://www.arduino.cc/en/Guide/ArduinoUno/, accessed on 9 April 2024), and a larger power supply would be required. 

#### 2.2.3. Thermistor

Temperature must be measured alongside water and air pressure as increases in temperature can proportionally increase air pressure, which influences water pressure. This is particularly important if deploying sensors over a larger spatial area (e.g., at the catchment scale in upland areas) due to elevation changes and linked local differences in climate. A 10 kΩ thermistor (B57863S0103F40; EPCOS, Summit House, London Rd, Bracknell RG12 2XH, UK) was chosen due to previously published compatibility, accuracy, and reliability in environmental monitoring [6], as well as reduced cost. To protect the thermistor, as well as the pressure sensor, from extreme currents, woody pieces, or sediment transport along the riverbed, the two components are housed in a small enclosure (RS PRO Grey ABS Enclosure, IP65, IK09, Grey Lid, 225 × 174.8 × 81.25 mm; RS PRO, RS Components Ltd., Birchington Road, Corby, Northamptonshire NN17 9RS, UK, Manufactured in Poland) that is filled with epoxy (RS PRO White Epoxy Potting Compound 250 g; RS PRO, RS Components Ltd., Birchington Road, Corby, Northamptonshire NN17 9RS, UK, Manufactured in UK) over the electrical elements for waterproofing. The differential pressure sensor’s two sensing ports extend into the water through two holes in this small enclosure to directly sense the water and air pressure. The thermistor is placed close to these holes. 

#### 2.2.4. Power Consumption

To limit power consumption, the Arduino-based logger was set to record every 60 min. The power supply to the Arduino-based logger is 4 × 1.5 V AA batteries (RS PRO Alkaline AA Battery 1.5V; RS PRO, RS Components Ltd., Birchington Road, Corby, Northamptonshire NN17 9RS, UK, Manufactured in China) chosen for their low cost and low environmental impact if exposed to water. Each battery supplies approximately 500 mAh toward the circuit, which is 2000 mAh in total. The Arduino-based logger current is 0.61 mA, which would allow for ~3300 h of logging when logging once every hour, equal to approximately 4.5 months of logging. The exact battery life varies depending on temperature, type of batteries used and their mAh capacity. 

### 2.3. Wiring and Coding

**Figure 2:**
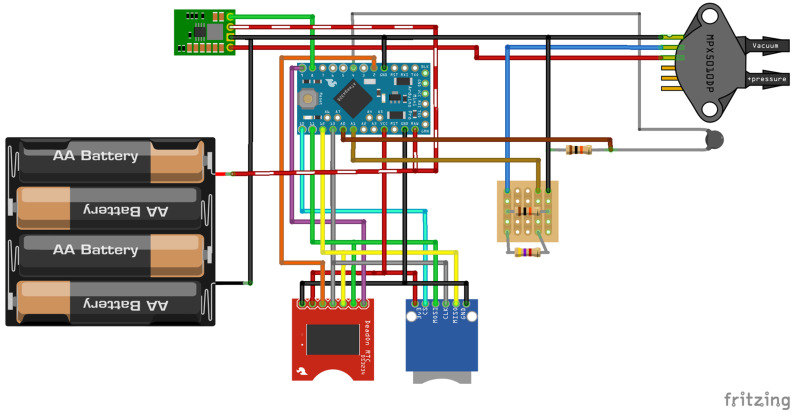
Schematic wiring diagram for differential pressure sensor. Generated through Fritzing.

**Figure 3:**
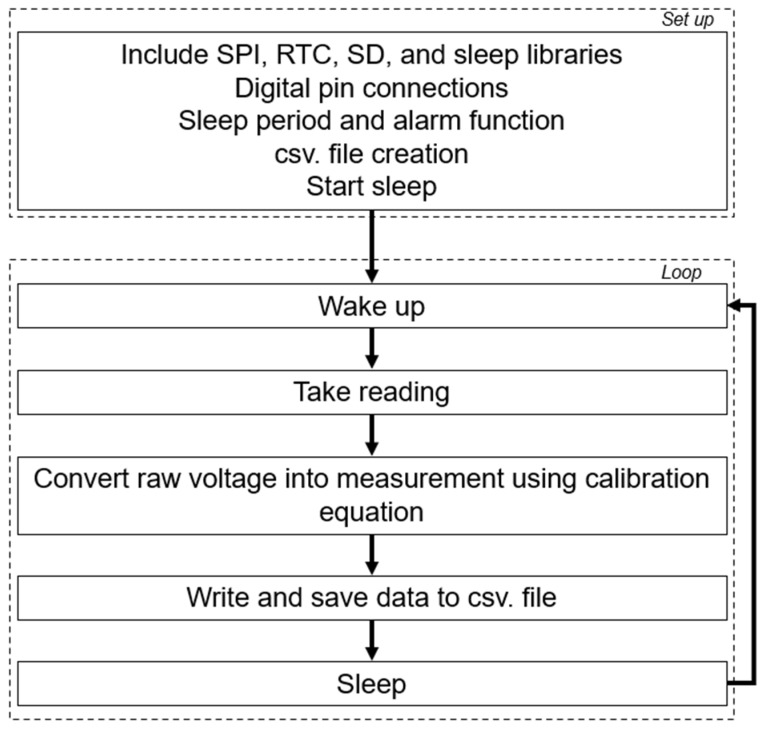
Simplified overview of code used to operate water depth logger. Code available at: https://github.com/rhpearce/waterdepthsensor (accessed on 9 April 2024).

The wiring for the sensor is provided in Figure 2 and a more explicit instruction guide for construction is available in the Supplementary Materials. The code is available at https://github.com/rhpearce/waterdepthsensor (accessed on 9 April 2024). An overview of the code’s structure and process is given in Figure 3.

### 2.4. Sensor Calibration

#### 2.4.1. Pressure

Once the sensor was constructed, a calibration for the 50 kPa pressure sensor was undertaken. A two-point calibration was deemed acceptable for such a short data range (estimating between 0 cm and 100 cm water depth at the deployment site). As water pressure (and voltage reading) is proportional to water depth, the calibration was conducted using a large tank of water reaching 78 cm depth (deepest available set up), and a ground point of 0 cm in the air. The sensor was positioned at points 0 and 78 cm for three minutes or until the voltage reading stabilised, from which the voltage reading was recorded. The two readings were plotted against water depth to obtain a linear equation of water depth against voltage. Based on the linear relationship between voltage and depth, the digital number reading by the Arduino can be used to convert voltage reading for pressure to actual water depth (Equation (1)). This process has been used in the construction and testing of other Arduino-based low-cost sensors [6].

*d* = *x* ∙ *P_dn* − *y* (1)

where *d* is water depth (cm), *P_dn* is the digital number read by the Arduino microcontroller, and *x* and *y* are the coordinates for the *y* intercept of the relationship between observed voltage (V) of the Arduino and water depth (cm) of the tank calibration.

The MPX5050DP has internal temperature compensation, which is factored into the voltage reading and calculation of depth relating to the thermal expansion of water, but it is also possible to use other pressure sensors, but temperature must be factored into the water depth calculation from pressure (Equation (2)),

(2) $$\rho \left(T\right)=\rho_{0}\cdot \;\left[1-\beta \cdot \left(T-T_{0}\right)\right]$$

where *ρ*(*T*) is the density of water at temperature *T*, $\rho_{0}$ is the density of water at a reference temperature, $T_{0}$, i.e., a calibration measurement, and β is the coefficient for volume expansion. Common values for water include [19]:

$$\begin{array}{l} \rho_{0}\;=\;999.972\mathrm{kg}m^{3}\\ T_{0}\;=\;3.98\;{}^{\circ}C\\ \beta \;=\;210\;\cdot \;10^{-6}K^{-1} \end{array}$$

#### 2.4.2. Temperature

Thermistor calibration followed a known calibration method [20] for a negative temperature coefficient derived from the Steinhart coefficients and based on the relationship between the resistance across the thermistor and the surrounding temperature. Where the thermistor was exposed to known temperatures at multiple intervals and the voltage reading recorded. The relationship between the voltage and the observed temperature produced a calibration equation to convert the voltage reading to actual temperature [20] (Equation (3))

(3) $$\frac{1}{T}=A+B\;\cdot \ln\left(R\right)+C{\left(\ln\left(R\right)\right)}^{3}$$

where *T* is the temperature in kelvin, *R* is resistance is ohms, and *A*, *B*, and *C* are the Steinhart–Hart coefficients that vary dependent on thermistor model and the temperature range of interest [20]. Temperature is then converted to degrees Celsius by subtracting −273.15.

### 2.5. Field Test Design 

**Figure 4:**
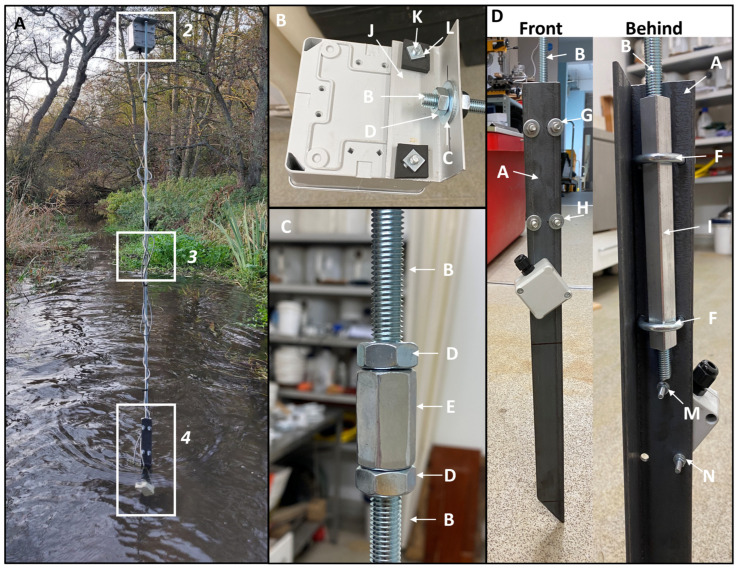
(**A**) Field deployment setup for the water depth sensors, showing: (**B**) the non-submersible logging component, which contains the Arduino, SD card, and RTC; (**C**) the threaded bar joint that can be repeated for height extension, and (**D**) the submersible sensor section attached to the secure angle iron foundation that has a sharpened end to anchor into river bed sediment, allowing the small enclosure containing the thermistor to sit on the river bed. See Table 2 for part names for corresponding letter labels.

The field test was conducted on the River Wissey, Norfolk, UK, a small, lowland chalk stream. In the deployment area, the channel width was 5 m with flows of <0.3 m^3^s^−1^ when initially deployed. The Wissey was chosen as a test location due to its narrow flow range (0.27 to 0.64 m^3^s^−1^) [21], which would not threaten equipment loss, and because it is a wadeable river, easy for logger installation. The deployment set up for the logger is shown in Figure 4. A section of angle iron was used as a base for installation, which was forced into the riverbed using a sledgehammer (Figure 4A,D). This section of angle iron already had the submersible part of the Arduino-based logger attached. Using the parts specified in Table 2, the installation infrastructure could be assembled in the field. Following the installation of the base, a threaded bar was held vertically in place using U-bolts (M6X33X25X18 U-Bolt Type-ABZP; Matlock, UK) (Figure 4D) and a joining nut (M10 joining nut; Easyfix UK, Galway, Ireland) was used to join two pieces of the threaded bar (1 m each) to extend the total height of the logger far above the maximum water level (Figure 4A). Threaded bar was chosen due to its flexibility, as it would bend slightly to allow access to the enclosure housing the SD card at the top of the threaded bar, which was attached using a bracket and nuts (Figure 4A,B). This flexibility also provides resilience against wind or any woody pieces that might enter the channel. The sensor was installed in the middle of the stream and deployed so that the joint of the angle iron faced upstream, with the enclosure containing the pressure sensor attached behind the joint, with the tube measuring water pressure pointing toward the riverbed and sitting as close to the river bed as possible. The depth from the centre of the small enclosure containing the pressure sensor to the riverbed was measured in order to adjust the data after deployment.

The logger was deployed for 6 months (*n* = 4485) to record a variety of hydrological and temperature conditions. The low-cost sensor was paired against a Solinst^®^ LevelLogger^®^ 5 Junior (Solinst Canada Ltd., 35 Todd Road, Georgetown ON L7G 4R8, Canada; https://www.solinst.com/products/dataloggers-and-telemetry/3001-levelogger-series/levelogger-junior/, accessed on 9 April 2024) as an industrial comparison (IC). This logger was chosen as an IC as it is the market equivalent of a low-cost sensor, matching in terms of capabilities to this study’s Arduino-based logger. The IC has a water level accuracy of ±0.1% FS, and water temperature accuracy of up to ±0.1 °C. The IC was connected to the lower U-bolt attached to the angle iron with a carabiner and wire to allow the logger to stand vertically in the groove of the angle iron and as close to the riverbed as possible. As well as the IC, a barometer was deployed to calculate the air pressure compensation post-test for the IC. All loggers sampled at 60 min intervals. Table 3 summaries some performance metrics of the Arduino-based logger and the IC.

Following deployment, comparison of performance via mean absolute error, root mean square error (RMSE), and a Bland–Altmann test [22,23] was conducted to assess the accuracy of the Arduino-based logger compared to the IC.

## 3. Results

**Figure 5:**
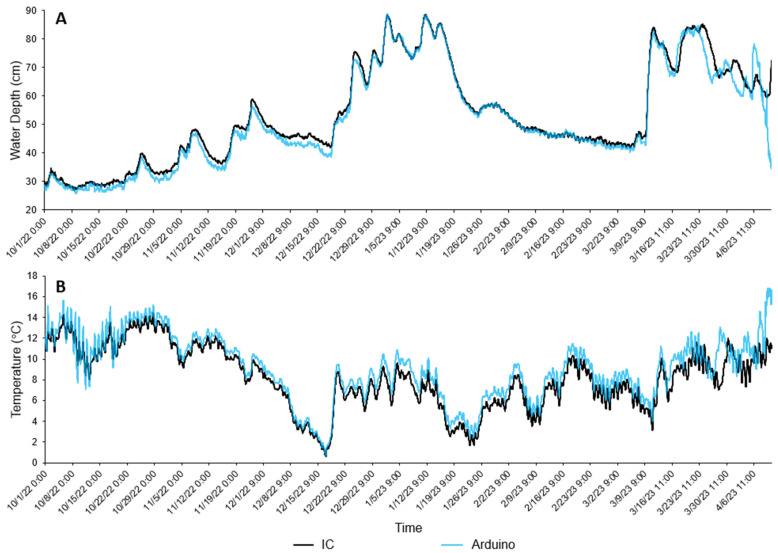
Logging period (1 October 2022–4 April 2023) of low-cost Arduino-based logger and industrial comparison logger (Solinst^®^ LevelLogger^®^ 5 Junior) visualising performance for (**A**) water depth (cm) and (**B**) water temperature (°C).

For the IC Solinst^®^ logger in the main channel there were 4614 readings between 00:00 on 1 October 2022 and 4:00 on 11 April 2023. Comparatively, the Arduino-based logger recorded 4485 readings, missing 129 readings between 1:00 on 23 November 2022 and 11:00 28 November 2022 due to a loss of power. Following a battery change on 28 November 2023, the Arduino-based logger continued to log until the end of the observation period. Figure 5A,B show the water depth and water temperature of the Arduino-based and IC loggers during the observation period. From the 10 March 2023 period, there is a clear drift that begins with Arduino-based logger that was determined to be due to undervoltage power supply toward the end of the battery life. Due to the reduced reliability of the data, they were excluded from the subsequent analysis with the new dataset focused on 1 October 2022 to 10 March 2023. Table 4 compares summary statistics between the Arduino-based and IC sensor for temperature and water depth. Comparing the reduced dataset (*n* = 3109), the water temperature data showed a parametric distribution, so applying a Pearson’s correlation coefficient, *r* = 0.992 (*p* ≤ 0.01). The water depth data were non-parametric, so a Spearman’s correlation coefficient showed *ρ =* 0.992 (*p* ≤ 0.01). 

### 3.1. Water Depth

Comparatively, the average depth for the Arduino-based logger was 48.01 ± 15.72 cm and 49.31 ± 15.34 cm for the IC. The coefficient of variation (CV) was 32.74%, which was higher than the IC at 31.01%. Mean absolute error for the Arduino-based logger comparative to the IC was 1.38 cm (or ±0.69 cm) with relative error at 0.031. The RMSE value was 1.69 cm. The low mean absolute error of ±0.69 cm would suggest that the error bias is within an acceptable range, dependent on application.

**Figure 6:**
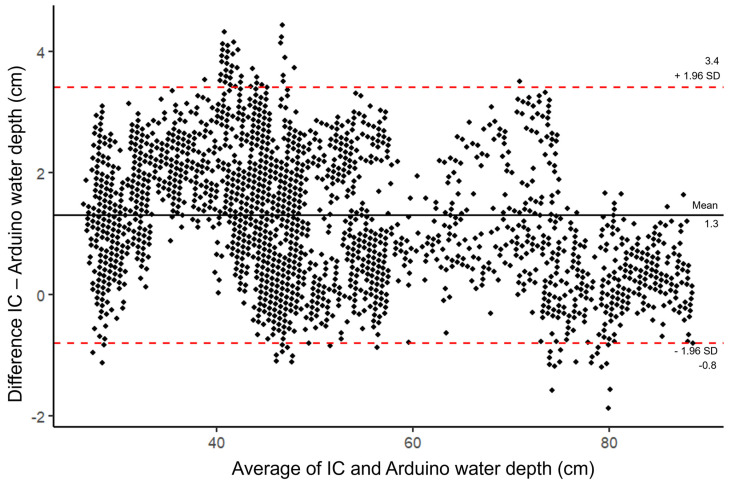
Mean difference in measurements between the industrial comparison (IC) and Arduino-based logger against the average measurement for each record of water depth (cm) and significant difference intervals (SD).

Figure 6 shows a Bland–Altman plot observing the systematic difference between the Arduino-based logger and the IC for water depth. The mean difference between the two loggers is 1.3 cm. The data, however, are unevenly distributed, but mostly concentrated between the significant difference intervals. Readings by the IC tend to be greater than the Arduino-based logger (Figure 5A), which suggests the Arduino-based logger consistently underestimates water depth. The underestimations are not consistent either, with greater underestimations around depths of 35–40 cm. Figure 6 also shows that between 60 and 80 cm, underestimation ranges become narrower and even begin to overestimate at the greatest depths. Based on Figure 6, the error in depth measurement for the Arduino-based logger would be greater at lower depths than at greater depths, but this skew towards greater error at lower depths is likely due to fewer measurements being higher depths as depths only exceeded 60 cm for a limited time (*n* = 686, 18.5% of logging period).

### 3.2. Temperature

**Figure 7:**
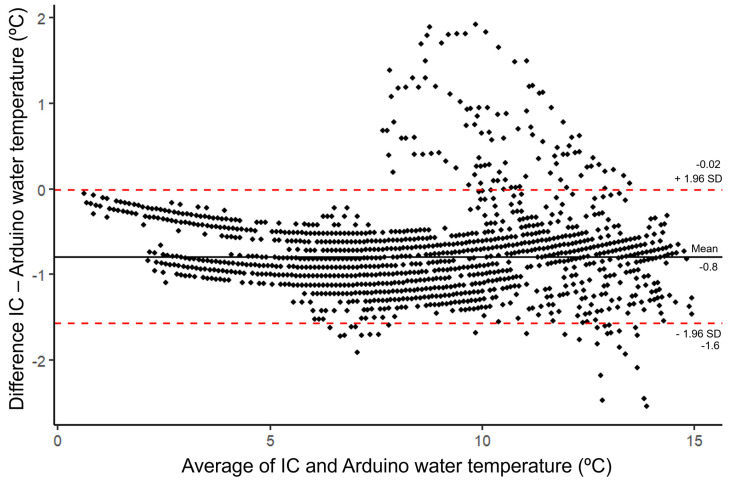
Mean difference in measurements between the industrial comparison (IC) and Arduino-based loggers against the average measurement for each record of water temperature (°C) and significant difference intervals (SD).

For water temperature, the Arduino-based logger shows a closer agreement to the IC: 8.82 ± 3.12 °C for mean water temperature compared to the IC with 8.02 ± 3.13 °C. The water temperature for the Arduino-based logger shows a lower CV at 35.37% compared to the IC at 38.95%. The mean absolute error for the Arduino-based logger’s water temperature readings is 0.83 °C (or ±0.415 °C), with a relative error of 0.12. The RMSE was 0.88 °C. The high correlation and low mean absolute error, RMSE, and relative error would evidence high accuracy for the Arduino-based logger, but Figure 5B and Figure 7 show some issues.

Figure 7 shows a Bland–Altman plot displaying the systematic difference between the IC and Arduino-based measurements for water temperature. Overall mean difference between the IC and Arduino-based loggers is low, −0.8 °C, which suggests that compared to the IC, the Arduino-based logger tends to overestimate. The range of the significant difference bounds is less compared to water depth, giving confidence that the methods are interchangeable. This could, however, relate to the narrower range of temperature data: 0.6 to 14.4 °C. Both significant difference bands are below zero, which would suggest that there is general trend for the Arduino-based logger readings to be greater than those of the IC. There seems to be a weak trend that overestimations are lower with lower temperatures. This trend is weakened, however, by a non-even distribution of points outside of the significant difference bounds above ~8 °C. This suggest that while some readings tend to overestimate within a predictable range, some readings, almost randomly overestimate and underestimate higher temperatures.

## 4. Discussion

Based on the field tests, the Arduino-based logger provided water depth and water temperature data, but there are definitive trade-offs with accuracy, costs, and feasibility, which, in its current form, limit its applications.

In terms of accuracy, the data show that, when comparing the Arduino-based logger with the IC logger, the Arduino-based logger was less accurate. Solinst^®^ state that the IC is accurate to ±0.5 cm, while the Arduino-based logger’s mean absolute error for water depth was ±0.69 cm. Similarly, for temperature, the Arduino-based logger was accurate to an average of ±0.415 °C. The overall accuracy of the Arduino-based logger is similar to the results of a low-cost ultrasonic sensor for groundwater monitoring based on an Arduino microcontroller, which found mean water depth of the low-cost sensor within 0.1 cm of the actual mean water depth [24]. To reduce the depth error, it is possible to improve the calibration process by including more staggered calibration intervals, which would improve variation in error between the calibration point extremes. Whether these deviations in accuracy are acceptable is clearly dependent on the study context. Research focusing on small-scale water depth fluctuations, whether in the field or under experimental conditions, may find the Arduino-based logger’s accuracy inadequate, but in the context of generating hydrological data to inform river management and restoration (e.g., frequency of river floodplain inundation events), a deviation of approximately 1.38 cm would be suitable for most instream monitoring, especially where research is undertaken over longer time periods and concerned with broader water depth changes linked to extremes (i.e., drought and flood periods) [25,26,27]. Ultimately, it is up to the individual and project to accept the accuracy capabilities of any piece of equipment, but it could be other factors, such as resource costs, which influence the decision to adopt low-cost monitoring technology and well as the clear advantage that the equipment can be adapted and improved upon by interfacing better quality sensors or shields with expanded capabilities depending on the needs of the local environment and the project

One key issue, as shown in Figure 5A,B, is exemplified best after 10 March 2023, there is a time drift with the Arduino-based logger for both temperature and water depth. Interestingly, this is not a delayed reading compared to the IC, which would suggest a time drift with the RTC (DS3234 DeadOn Real Time Clock (RTC) Breakout Board—BOB-10160; 6333 Dry Creek Parkway, Niwot, CO 80503, USA), but an earlier reading. From further inspection of the data, it is likely that a combination of power loss from the batteries and an issue with the RTC are responsible. It is suspected that during the deployment period, the batteries drained to a point where the power supply was under the required voltage to operate the logger components, which affected the time keeping capabilities of the RTC. The exposure of the electronics to a moist environment could have also impeded the RTC’s performance, as there was visible corrosion on the electronics following deployment. Further, it should be noted that this Arduino-based logger was deployed as part of a wider project and so had been in the field since November 2021 (it was retired in June 2023), suggesting a logger shelf life, in river settings, of just under 2 years. The logging failure here could indicate the start of this decline in data quality.

One clear advantage of the Arduino-based logger compared to the IC Solinst^®^ logger is cost. The Solinst^®^ costs £408 for one logger (accurate as of 19 January 2022, equivalent to USD 514.83 at time of publication), but requires a barometer for atmospheric compensation (£288, equivalent to USD 363.41 at time of publication) and field reader to download the data (£117, equivalent to USD 147.63 at time of publication), which increases overall costs for the deployment of one water depth station to £813 (equivalent to USD 1025.87 at time of publication). The Arduino-based logger costs approximately £133.35, equivalent to USD 168.26 at time of publication (if including this project’s logger installation method, £109 for the logger itself, equivalent to USD 137.54 at time of publication) as of autumn 2023, but this does not include the costs of development, building, and testing, which are all incorporated into the Solinst^®^ logger costs. If this extra time and labour was accounted for, the price for one Arduino-based logger would be much higher. This cost, however, would decrease over time as production procedures became more efficient and established with costs per logger also decreasing due to discounts from individual parts sold in batches. Compared to other low-cost hydrological monitoring projects, the cost for this Arduino-based logger is similar to other studies. For example, a surface velocity sensor using Doppler radar was developed with an accuracy of 0.07 m/s and a cost of <USD 50 (~£39 at time of publication) [12]. Further, a study used timelapse photography to monitor water depth at a gauging station recording 33 runoff events over a two year period in an urban stream near Rio Rancho, New Mexico, costing < USD 200 (~£157 at time of publication) [28]. The explorative, trial-and-error nature of low-cost environmental sensors is well established in the literature and this, along with accuracy limitations (as dependent on the low-cost sensor used), is a recognised barrier to the use of the technology in real-life monitoring scenarios or other research settings [6]. Comparable to the findings made here, studies comparing the performance of different low-cost environmental sensors, including a sediment trap, a water quality sensor, multi-parameter weather stations, and a sonic anemometer for monitoring sand transport, showed similar drawbacks associated with accuracy and uncalculated labour costs [6]. For example, an Arduino-based water depth using a 2-bar pressure sensor (TE Connectivity MS5803-02ba; https://www.te.com/usa-en/product-CAT-BLPS0010.html, accessed on 9 April 2024) compared to an industrial standard found the mean absolute error to be 1.46 cm and had an RMSE of 1.88 cm [6], both greater than the error calculated for this low-cost Arduino-based water depth sensor.

Once a manufacturing and testing process is established for a new low-cost sensor, distinct advantages are offered. If established accuracy bands are acceptable, then low-cost sensors can provide an adaptable and scalable environmental logging system applicable to various environmental investigations. Due to their low costs, more loggers can be deployed in a given study area, thus increasing the spatial resolution of the data collection. This capability is becoming increasingly important with the need for science and monitoring studies at larger landscape and catchment scales [27]. Similar to the IC loggers, Arduino-based loggers also have the advantage of collecting data at a high temporal resolution; although, as observed in this study, the Arduino water depth sensor had a problem with power supply and this reduced its useful timespan. Despite initial calculations putting battery life at 4.5 months, the 4 × AA batteries were enough to last for 3 months only, which would be less if the logging interval was less than 60 min. This short effective deployment time requires site visits to change batteries, which, combined with the aforementioned construction time and labour costs, is neither budget-friendly nor sustainable. The power loss observed in November 2022, which resulted in the loss of 129 readings, cannot be proven, but is assumed to be related to the low-cost elements and the electronics performing in cold and damp conditions, which can interfere with the circuitry causing power loss through the system. This was determined as batteries and components had visible corrosion and, after deployment, power output from the batteries was 0 V. Thus, compared to the Solinst^®^ IC, which has a battery life of 5 years reading once per minute, the Arduino-based logger seems more like a resource drain than an advantage, especially if many loggers are included in a study. To overcome this issue, more renewable power supply options need to be considered. For example, solar panels would be appropriate for open floodplain water depth loggers, and the turbines used on a low-cost flow meter could also function as power supply. Further, more investment in waterproofing and field testing ideally needs to be considered to overcome data loss.

A specific advantage of “build-it-yourself” sensors is the prospect of continual development. Based on the results of this field test, the Arduino-based logger could and should undergo further development and a way forward can be suggested. In terms of hardware, a 3G/GSM communication module could be added to the design to allow the logger to send data in situ, reducing the need for site visits to download data. In combination with a solar panel, the battery life could be extended, and this would further reduce the need for visits while also increasing the logger’s sustainability. In terms of accuracy, based on this field test, a post-deployment calibration could be undertaken using the relationship of variation between the Arduino-based and the IC logger, or the Arduino-based readings could be adjusted using the mean difference for each parameter [23]. These developments would come with increased resource costs (requiring a cellular enabled MCU and a USB solar panel adding ~£25 to the build cost), and data handling (e.g., understanding of online databases and webhook) time, however. Another small improvement for the Arduino-based logger is to use 1.7 kΩ and 3.3 kΩ resistors for the voltage divider instead of 10 kΩ and 4.7 kΩ. The two new suggested resistors are a standard way of achieving max 3.3 V output from 5 V, which would be safer to use, but this does reduce precision below maximum pressure readings. Considering the advantages of other microcontrollers over the chosen Arduino Pro Mini 3.3 V could also improve this low-cost water depth sensor. For example, the inclusion of the ESP 32 (Espressif Systems, https://www.espressif.com/en/products/socs/esp32, accessed on 9 April 2024), which has been incorporated into an Arduino Nano, would provide Wi-Fi and Bluetooth capabilities that could assist in data collection.

Reviewing the performance and limitations of the Arduino-based logger, it is clear that it is not appropriate for all monitoring situations. For example, under the current design, the Arduino-based logger could not persist in a long-term hydrological study as part of an analysis on long term variability [29,30], which is crucial to understanding the response of hydrological systems under climate change. With improvements to the design, such as waterproofing and power source, the Arduino-based logger’s life span could be expanded, but compared to some industry standard loggers, which have greater expense, but increased life span, negate the low-cost advantage. Another key improvement for long-term deployment would be to use a stainless-steel base instead of iron as even during this short-term deployment there was noticeable corrosion, which would weaken the overall structure over time. There are potential uses for these loggers, however, such as ground-truthing hydrological models in a validation period or data collection in headwaters where there has been no previous data collection.

## 5. Conclusions

Overall, the Arduino-based water depth sensor combines low-cost electronics, housing, and software to produce a relatively accurate sensor compared to the IC. The logger, therefore, is not applicable in all research or monitoring settings due to issues with accuracy, but budgets and research standards permitting, could assist in the collection of baseline data that could direct and inform future study. The hidden costs of research and development of “build-it-yourself” loggers would suggest that this is only applicable in certain scenarios, such as for university research or citizen science programmes [7,31]. The role, therefore, of low-cost monitoring solutions in wider environmental monitoring schemes (especially long-term monitoring) is questionable, but the high spatial and temporal resolution of the data that can be collected as well as microcontrollers themselves have potential in the development of low-cost sensors. Indeed, future developments in terms of technical accuracy, capabilities, design, and setup could shift the balance and make low-cost sensing systems more attractive.

## Figures and Tables

**Table 1:** Parts and price list. Parts inclusive of those used in construction in 2021, whereas price is accurate as of autumn 2023. See https://docs.google.com/spreadsheets/d/1xHk34M-YiePO2RZdtJb8Tk1eWIesMuvv/edit?rtpof=true&sd=true for links (accessed on 9 April 2024).

Part Name	Quantity	Cost/Item (£)	Total Cost (£)
Arduino pro mini 3.3 V	1	4.90	4.90
Micro SD card 1 GB	1	4.28	4.28
Real-time clock (RTC) module DS3234	1	20.10	20.10
Micro SD card holder 3.3 V	1	0.13	0.13
10 kΩ thermistor	1	3.94	3.94
MPX5050DP differential pressure sensor 50 kPa	1	23.66	23.66
PCB screw terminals (2-terminal)	12	0.21	2.52
Battery holder, coil spring (4 × AA)	1	1.26	1.26
AA Batteries	4	0.28	1.12
Battery snap connector	1	0.69	0.69
4-core cable (~1.5 m)	1	0.75	0.75
Cable glands (4 mm minimum, IP68)	2	1.05	2.10
Rubber tubing (3 mm × >1 m)	1	0.77	0.77
Small enclosure	1	3.27	3.27
Larger enclosure	1	13.10	13.10
Epoxy (250 g)	1	6.83	6.83
10 kΩ 0.4 W ± 1% resistor	2	0.13	0.26
4.7 kΩ 2 W ± 5% resistor	1	0.108	0.108
5 V step up regulator	1	12.80	12.80
Insulated wire	1	0.29	0.29
CH340 USB to TTL serial adapter	1	2.99	2.99
Jumper wires—connected 6″ (F/F, 20 pack)	1	1.72	1.72
Arduino Pro Mini V1 PCB Design	1	5.47	5.47
		**Total**	**113.05**

**Table 2:** Parts and price list for suggested sensor installation. Parts inclusive of those used in 2021 construction, whereas price is accurate as of autumn 2023. Label corresponds to annotations in Figure 4. * Needs to be adapted depending on the enclosures used with bolts often coming with the enclosures.

Part Name	Label	Quantity	Cost/Item (£)	Total Cost (£)
Angle iron (40 mm × 40 mm × 3 mm) 1 m	A	1	4.17	4.17
M10 steel threaded bar (1000 mm)	B	2	3.40	6.80
M10 × 2.5 mm washers	C	2	0.11	0.22
M10 nuts	D	2	0.06	0.12
M10 joining nuts	E	1	0.57	0.57
M6 U-bolts	F	2	1.04	2.08
M6 hex nuts	G	4	0.08	0.33
M6 × 1.6 mm washers	H	4	0.07	0.27
Angle aluminium (12.7 mm × 12.7 mm × 3.2 mm) 20 cm	I	1	0.36	0.36
Angle aluminium (101.6 mm × 101.6 mm × 3.2 mm) 20 cm	J	1	4.80	4.80
M8 bolts	K	2	0.11	0.22
M8 × 2 mm washers *	L	2	0.09	0.18
M4 nuts *	M	2	0.05	0.10
M4 × 1 mm washers *	N	2	0.04	0.08
			**Total**	**20.30**

**Table 3:** Summary of known performance metrics between the Arduino-based logger and the IC. * Information from Solinst^®^. ^ Calculation is of potential capacity of 1 GB SD card after deployment resulted in 54 KB of readings.

**Performance Metric**	**Arduino-Based Logger**		Industrial Comparison *	
Operating voltage	5 V		NA	
Power supply	4 × AA batteries		NA	
Estimated battery life	4.5 months at 1 reading per hour		5 years at 1 reading per min.	
Memory	~33.3 million ^		75,000 data points	
Cost	£113.05		£408	
Clock accuracy	±3.5 ppm from −40 to +85 °C (±184 min/yr)		±1 min/year (−20 to 80 °C) (0.0190258 ppm)	
**Sensor Specific Metric**	**Water Depth**	**Temperature**	**Water Depth**	**Temperature**
Accuracy			±0.5 cm	±0.1 °C
Data range	<5.09 m	0 to +70 °C	<5 m	−20 to 80 °C

**Table 4:** Summary statistics comparing performance of Arduino-based logger and industrial comparison sensors at recording water depth.

	Arduino-Based Logger		Industrial Comparison	
	Water Depth (cm)	Temperature (°C)	Water Depth (cm)	Temperature (°C)
Mean	48.01	8.82	49.31	8.02
Median	44.99	8.69	46.2	7.8
SD	15.72	3.12	15.34	3.13
CV (%)	32.74	35.37	31.01	38.95

## Data Availability

Data are contained within the article and Supplementary Materials.

## Supplementary Materials

The following supporting information can be downloaded at: https://github.com/rhpearce/waterdepthsensor (accessed on 9 April 2024).
